# Evaluation of Accuracy and Safety of the Next-Generation Up to 180-Day Long-Term Implantable Eversense Continuous Glucose Monitoring System: The PROMISE Study

**DOI:** 10.1089/dia.2021.0182

**Published:** 2022-01-31

**Authors:** Satish K. Garg, David Liljenquist, Bruce Bode, Mark P. Christiansen, Timothy S. Bailey, Ronald L. Brazg, Douglas S. Denham, Anna R. Chang, Halis Kaan Akturk, Andrew Dehennis, Katherine S. Tweden, Francine R. Kaufman

**Affiliations:** ^1^ University of Colorado, Aurora, Colorado, USA.; ^2^ Rocky Mountain Diabetes Center, Idaho Falls, Idaho, USA.; ^3^ Atlanta Diabetes Associates, Atlanta, Georgia, USA.; ^4^ Diablo Clinical Research, Walnut Creek, California, USA.; ^5^ AMCR Institute, Escondido, California, USA.; ^6^ Rainier Clinical Research Center, Renton, Washington, USA.; ^7^ Clinical Trials of Texas, Inc., San Antonio, Texas, USA.; ^8^ John Muir Health, Concord, California, USA.; ^9^ Senseonics, Inc., Germantown, Maryland, USA.

**Keywords:** Continuous glucose monitoring, Implantable sensor, PROMISE study, Eversense

## Abstract

***Background:*** Use of continuous glucose monitoring (CGM) systems is being rapidly adopted as standard of care for insulin-requiring patients with diabetes. The PROMISE study (NCT03808376) evaluated the accuracy and safety of the next-generation implantable Eversense CGM system for up to 180 days.

***Methods:*** This was a prospective multicenter study involving 181 subjects with diabetes at 8 USA sites. All subjects were inserted with a primary sensor. Ninety-six subjects had a second sensor, either an identical sensor or a modified sensor (sacrificial boronic acid [SBA]), inserted in their other arm (53 and 43 subjects, respectively). Accuracy was evaluated by comparing CGM to YSI 2300 glucose analyzer (Yellow Springs Instrument [YSI]) values during 10 clinic visits (day 1–180). Confirmed event detection rates, calibration stability, sensor survival, and serious adverse events (SAEs) were evaluated.

***Results:*** For primary sensors, the percent CGM readings within 20%/20% of YSI values was 92.9%; overall mean absolute relative difference (MARD) was 9.1%. The confirmed alert detection rate at 70 mg/dL was 93% and at 180 mg/dL was 99%. The median percentage of time for one calibration per day was 56%. Sixty-five percent of the primary sensors survived to 180 days. For the SBA sensors, the percent CGM readings within 20%/20% of YSI values was 93.9%; overall MARD was 8.5%. The confirmed alert detection rate at 70 mg/dL was 94% and at 180 mg/dL was 99%. The median percentage of time for one calibration per day was 63%. Ninety percent of the SBA sensors survived to 180 days. No device- or insertion/removal procedure-related SAEs were reported.

***Conclusion:*** These data show the next-generation Eversense CGM system had sustained accuracy and safety up to 180 days, with an improved calibration scheme and survival, using the primary or SBA sensors.

## Introduction

Real-time continuous glucose monitoring (CGM) systems have been shown to be useful tools to lower and/or maintain hemoglobin A1c (HbA1c) and lower time spent in hypoglycemia in people with diabetes, who are insulin treated.^1–3^ There are sufficient data provided by CGM to enable the user to make diabetes management decisions concerning dosing for meals, preventing and correcting hypoglycemia and hyperglycemia, and exercising. In addition, analysis of real-time CGM data allows patients and their health care providers to analyze patterns and trends to alter aspects of the diabetes regimen, as well as determine if established targets for glucose levels, such as time in range and percentage of time spent above and time spent below range, are being met.^2^ However, barriers to adherence to CGM remain so that adoption, although increasing, is still not optimal and intermittent use or discontinuation of CGM remains a clinical problem.^4–6^

Of the currently approved CGM systems, only the Eversense CGM system is a long-term implantable CGM system labeled for use up to 90 days in the USA and up to 180 days in Europe.^7–9^ The Eversense sensor, designed to be inserted in the upper arm as previously described,^8^ was intended to decrease the burden of repeated transcutaneous sensor insertions every 7, 10, or 14 days, with the goal to improve adherence and acceptance of CGM by a wider range of insulin-treated patients. In clinical trials, Eversense system wear times (transmitter on top of the skin generating glucose measurements) have exceeded 95%^7–9^ and in the real-world analysis postregulatory approvals, median wear time was 83%.^10^ Importantly, both wear times exceed the recommended time of 70% for diabetes management and data analysis^2^ and the suggested time of 80% as a criterion to enter clinical trials, which assess CGM effectiveness.^4,11,12^

There are many unique attributes to the Eversense CGM system beyond its long duration and insertion by a health care provider.^7–9^ The Eversense CGM system measures glucose every 5 min through a fluorescent-based optical methodology with the glucose information sent directly to a Mobile Medical Application (MMA) app on the smartphone. The system has high accuracy with a mean absolute relative difference (MARD) of 8.5%.^8^ There is no interference with glucose measurements from Vitamin C or acetaminophen commonly seen with some of the available CGM systems. The transmitter, placed on the skin over the sensor, powers the sensor and calculates the glucose values. The transmitter can be removed without sacrificing the sensor, it allows for on-body vibratory alerts for hypoglycemia and hyperglycemia and is held in place with a mild silicone-based adhesive associated with low rates of skin irritation^7–10^ as it is replaced every 24 h.

The purpose of the PROMISE study was to evaluate the next-generation Eversense system for up to 180 days in subjects with diabetes who were 18 years of age or older. The data were analyzed after study completion to evaluate algorithm modifications, which were primarily designed to reduce the number of calibrations required over the lifetime of the sensor. In addition, a subset of subjects had a second sensor (sacrificial boronic acid (SBA) sensor) inserted with a specific modification to the glucose-binding indicator chemistry, designed to enhance longevity by reducing oxidation, which can limit the effectiveness and duration of implanted optical sensors.

The interferants of the original sensor were previously shown to be (1) intravenously administered mannitol or sorbitol or those components administered as an irrigation solution of peritoneal dialysis solution and (2) the tetracycline class of antibiotics.^13^ Substances may interfere with the Eversense sensor by causing an increase in the glucose signal by binding to the glucose-indicating hydrogel or emitting light within the spectral operating range of the sensor, or substances may reduce signal by absorbing excitation light or by absorbing or quenching hydrogel fluorescence.

In vitro analytical assessment of the primary sensor compared to the SBA sensor demonstrated comparable results regarding fluorescence signal, glucose modulation, and glucose binding (data not shown). The comparability of these parameters between the primary sensor and SBA sensor, combined with the knowledge of the mechanisms by which substances can potentially interfere with glucose measurements in the fluorescent hydrogel-based Eversense sensor, led the developers to conclude that previously collected interference data are appropriate for the SBA sensor. Specifically, because there was no change to the glucose binding indicator molecule, it was deduced that the binding kinetics with any interfering substance would be unaffected.

This article describes the performance and safety results of the PROMISE study, including both the primary Eversense sensor with the standard indicator chemistry, as well as the modified SBA sensors. The performance assessments included evaluating the accuracy and longevity of the two sensor configurations.

## Methods

### Study design and participant enrollment

PROMISE (NCT03808376) was a prospective, multicenter, unblinded, nonrandomized study involving subjects with type 1 (T1D) and type 2 (T2D) diabetes at eight sites in the United States. It was designed to assess the safety and accuracy of the next-generation Eversense CGM system for up to 180 days. The study was conducted between December 27, 2018, and May 08, 2020, and included both in-clinic visits and home use of the system.

Individuals were eligible for participation if they were 18 years of age or older and had clinically confirmed T1D or T2D for at least 1 year. Individuals were excluded from participation if they had any of the following: a history of unexplained severe hypoglycemia or diabetic ketoacidosis, necessitating an emergency room visit or hospitalization during the previous 6 months; a condition complicating sensor placement, operation, or removal; symptomatic coronary artery disease, unstable angina, myocardial infarction, or stroke in the previous 6 months; uncontrolled hypertension; hematocrit <30% or >50%; and lactation or pregnancy during the study. The study was performed in accordance with the Declaration of Helsinki and was approved by centralized internal review boards. All participants provided both verbal and written informed consent.

### Study device

The Eversense CGM system consists of an implantable fluorescence-based cylindrical glucose sensor (3.5 × 18.3 mm), a smart transmitter, and MMA (app) that displays glucose data in real-time and operates on a mobile device, which have been described previously.^8,9^

During the study, modified sensors were evaluated. These sensors had a minor change in the glucose-binding indicator hydrogel, consisting of the inclusion of the 4-vinylphenylboronic acid (VPBA) monomer. The VPBA acts as an SBA that serves as a target for reactive oxygen species (ROS) and subsequently protects the boronic acid moiety that participates in the glucose-binding reaction used to determine interstitial glucose values.

Ninety-six of 181 subjects had two sensors inserted, and the secondary sensors had the glucose values blinded to the users. Fifty-three of the secondary sensors inserted were identical to primary sensors for analysis of precision, while the other 43 of the secondary sensors were SBA sensors. The analysis for the primary sensors and SBA sensors utilized a glucose calculation algorithm that assessed changes in glucose-binding sensitivity.

### Study procedures

The accuracy of the next-generation Eversense 180-day CGM system was evaluated during in-clinic visits comparing CGM glucose values and plasma glucose values measured after centrifugation of whole blood samples collected in tubes containing ethylenediaminetetraacetic acid at 8500 rpm using the Yellow Springs Instrument (YSI) (2300 Stat Plus Glucose and Lactate Analyzer; Yellow Springs, OH) bedside glucose analyzer. Study visits occurred for baseline screening, sensor insertion (day 0), and accuracy visits on day 1, 7, or 14, 22, 30, 60, 90, 120, 150, and 180 (also sensor removal) for sampling of up to 10 h. Samples were drawn every 15 min, except when reference glucose was ≤70 or ≥300 mg/dL, at which time sampling was every 5 min. Each reference glucose value was paired to the corresponding CGM glucose obtained within 5 min after the reference blood draw.

To test sensor performance across the entire reporting range of the device (40–400 mg/dL), data were collected in the hypoglycemic and hyperglycemic ranges by having eligible subjects on insulin participate in either hypoglycemia or hyperglycemia challenges at each visit. Subject's glucose levels were artificially raised using mixed meals of 30%–40% carbohydrate content or lowered using subcutaneous insulin dosing based on each participant's individualized insulin sensitivity under close provider's supervision.

At the baseline screening visit, investigators obtained participant demographics and medical history and performed laboratory measurements (i.e., HbA1c and hematocrit), a physical examination, and an electrocardiogram. Urine pregnancy testing was also conducted in female participants. Sensors were inserted into the upper arm at the insertion visit (day 0) by trained providers (i.e., physicians, nurse practitioners, or physician assistants). At all visits after baseline screening, investigators assessed serious adverse events (SAEs) and adverse events (AEs), sensor insertion sites, hematocrit levels, pregnancy status, and changes in medications. Details of the sensor insertion procedure have been previously described.^8^

The participant was prompted to begin calibration 24 h after insertion. Transmitter(s) were worn over the sensor(s) and participants were prompted for calibration entry by the transmitter's vibration, as well as smartphone app. The CONTOUR Next One, blood glucose (BG) monitoring system (Ascensia Diabetes Care, NJ), and respective test strips were used to calibrate the CGM. The BG meter data were downloaded at each follow-up visit. Participants and investigators were able to see CGM values, including all alerts and prompts from the app for the duration of the study with the primary sensor; however, participants were informed to make all diabetes care decisions based on current clinical standards of care using BG meter data.

During the study, if calibration was overdue by 4 h after it was required, the system stopped displaying glucose values and if overdue by 12 h, the system required reinitialization to show data to the user, which required four fingersticks within 24 h. Note that study participants were requested to calibrate twice per day during the study. The data were reanalyzed after the study was conducted to determine frequency of calibration needed every 24 h (one or two calibrations).

Participants maintained their routine diabetes treatment throughout the visits. HbA1c levels were obtained at day 90 and 180 visits. The sensors were removed after the day 180 visit with the procedure previously described.^8^ Participants returned ∼10 days after removal for follow-up to assess the healing of the removal site.

### Study outcomes

#### Accuracy

Device performance was primarily evaluated based on how the sensor glucose measurements compared with a YSI glucose measurement. Mean and median absolute relative difference (MARD) and system agreement for all paired sensor and reference measurements through ∼180 days postinsertion was calculated for CGM glucose values within 40–400 mg/dL for the accuracy evaluations. The percent of sensor readings within 15 or 20 mg/dL for YSI values ≤80 mg/dL and 15% and 20% for YSI values >80 mg/dL was used to calculate all 15%/15% and 20%/20% agreement rates.

#### Alert rates

Confirmed event detection rates and true alert rates were calculated for hypoglycemic and hyperglycemic events. When a hypoglycemic (low alert 60 and 70 mg/dL) or hyperglycemic (high alert 180 and 240 mg/dL) event occurred with the reference YSI value, a confirmed event detection meant the CGM measurement or predicted CGM measurement reached the alert threshold within ±15 min. In contrast, a true threshold alert occurred when a CGM measurement or predicted CGM measurement reached the hypoglycemic or hyperglycemic alert threshold and at least one reference YSI measurement within ±15 min also reached beyond the same alert threshold.

#### Sensor longevity

Kaplan–Meier survival curves were used to describe sensor longevity.

#### Calibration stability

Agreement between CGM and YSI measurements was assessed over the duration between calibration entries by stratifying matched pairs data in 2-h increments over the period of 0–28 h postcalibration. The effectiveness of using one or two calibration points per day was evaluated by assessing the performance of the CGM system spanning the duration between calibration points. When the rate of change in sensor signal sensitivity showed a predicable stability for a 24-h period, the system shifted to prompt the user for one calibration per day, starting in the third week of system use.

#### Precision

Of the 53 subjects with identical primary and secondary sensors (two primary chemistry sensors inserted), the between-sensor precision was characterized based on the paired CGM system readings from the primary and secondary sensors worn simultaneously. Imprecision was measured by paired ARD (PARD) and percent coefficient of variation (PCV).

#### Safety

The safety endpoint was the rate of device-related or sensor insertion/removal procedure-related SAEs throughout the study, including sensor removal and final follow-up visit. The incidences of all procedure-related, device-related AEs and all AEs regardless of relatedness were assessed during all in-clinic sessions and home use. All reported AEs were adjudicated by an independent medical monitor for relatedness to the device, sensor insertion/removal procedures, and study procedure, which included those that occurred during the hyperglycemia and hypoglycemia challenges. AE severity was graded by the site principal investigator.

### Statistical analysis

Descriptive analyses were performed to evaluate the accuracy of the CGM system over time and glucose range, concurrence of system readings in comparison to the YSI reference, and alert performances. The proportion of patients experiencing at least one device-related or insertion/removal procedure-related SAE over the operating life of the sensor was determined along with the associated exact 95% confidence interval. Incidences of other safety analyses were tabulated.

## Results

### Subjects

Two hundred eight subjects were enrolled, and 181 subjects were inserted with sensor(s); 85 subjects were inserted with one sensor only and 96 subjects were inserted with two sensors, one in each arm. Reasons for not proceeding to sensor insertion were related to screen failure in 25 subjects and 2 subjects withdrawing after screening due to inability to comply with the study visit schedule. One hundred seventy subjects (94%) completed day 180. Ten subjects withdrew from the study (5.5%) after sensor insertion (four of these subjects withdrew before day 90 and six subjects withdrew before day 180). There were no subject terminations for safety reasons. Participant baseline characteristics are presented in Table 1.

**Table 1.** Baseline Participant Characteristics

Demographic	Value
Gender, *n* (%)	
Male	85 (47.0)
Female	96 (53.0)
Age (years)^a^	48.6 (14.9)
Min, max	18, 77
Ethnicity, *n* (%)	
Hispanic	23 (12.7)
Non-Hispanic	158 (87.3)
Race, *n* (%)	
Caucasian	163 (90.1)
Black or African American	10 (5.5)
Asian	4 (2.2)
American Indian or Alaska native	2 (1.1)
Native Hawaiian or other Pacific Islander	0 (0.0)
More than one race Self-identified	2 (1.1)
Body mass index (kg/m^2)a^ Min, max	31.4 (7.2) 19.0, 61.0
Normal (<25 kg/m^2^), *n* (%)	28 (15.5)
Overweight (≥25 and <30), *n* (%)	53 (29.3)
Obese (≥30), *n* (%)	100 (55.3)
Diabetes history	
Years since diabetes diagnosis (years)^a^	22.0 (13.3)
Min, max	1, 56
Diabetes type, *n* (%)	
Type 1	126 (69.6)
Type 2	55 (30.4)
Type of insulin therapy, *n* (%)	
None (oral diabetes medications only)	16 (8.8)
Multiple daily injections	65 (35.9)
Continuous insulin infusion pump	92 (50.8)
Other (basal only or 1 injection per day)	8 (4.4)
HbA1c (%)^a^ Min, max	7.6 (1.3) 4.8, 12.6

^a^ Mean (SD).

HbA1c, hemoglobin A1c; SD, standard deviation.

### The primary sensor results

#### Accuracy

A total of 49,613 matched glucose pairs were collected. Table 2 shows the overall accuracy data, as well as the data by glucose ranges. The overall MARD over the glucose range of 40–400 mg/dL was 9.1%. The percent of sensor readings within 15%/15% was 85.6% and the 20%/20% agreement rate was 92.9% over the duration of the study (up to 180 days).

**Table 2.** Continuous Glucose Monitoring System Agreement to Reference Within Yellow Springs Instrument Glucose Ranges Through 180 Days: Primary Sensor

YSI glucose range (mg/dL)	No. of paired CGM and YSI reference points	Mean percent 15%/15% of reference	Mean percent 20%/20% of reference	MARD*^a^* (%)	Median absolute relative difference (%)
Overall	49,613	85.6	92.9	9.1	6.7
40–60	2281	83.2	89.4	9.4	7.0
61–80	5270	84.1	92.2	8.8	7.0
81–180	19,001	82.7	90.9	9.0	6.7
181–300	14,578	87.9	94.7	7.7	5.9
301–350	6862	90.6	96.5	7.1	5.9
351–400	1510	87.8	93.9	8.0	6.3

^a^ MAD (mg/dL) was calculated for glucose values ≤80 mg/dL.

CGM, continuous glucose monitoring; MAD, mean absolute difference; MARD, mean absolute relative difference; YSI, Yellow Springs Instrument.

The accuracy data across the lifetime of the sensor, from day 1 to 180 after insertion, are given in Table 3. The system accuracy was lowest on day 1 (80% within 15%/15%) and was improved thereafter, consistent with the trend observed across CGM systems.

**Table 3.** Continuous Glucose Monitoring System Accuracy by Visit: Primary Sensor

Day number	Number of paired CGM and YSI reference points	Mean percent 15%/15% of reference	Mean percent 20%/20% of reference	MARD*^a^* (%)	Median absolute relative difference (%)
Day 1	5584	80.0	89.0	11.0	8.0
Day 7	2724	83.1	91.3	9.6	7.2
Day 14	2318	83.1	91.7	9.2	6.8
Day 22	6198	85.3	93.6	9.1	6.9
Day 30	6488	88.4	94.8	8.4	6.1
Day 60	6345	90.5	95.8	7.7	6.0
Day 90	6039	88.7	94.4	8.2	6.2
Day 120	5173	85.5	93.3	9.2	6.7
Day 150	4227	85.5	92.7	9.6	6.9
Day 180	4517	81.0	89.6	10.4	7.5

^a^ MAD (mg/dL) was calculated for glucose values ≤80 mg/dL.

#### Alert rates

Detection rates confirming a hypoglycemic or hyperglycemic event showed the confirmed event detection rate at the alert setting of 60 and 70 mg/dL were 87% and 93%, respectively, and at 180 and 240 mg/dL were 99% and 98%, respectively. The true alert rate at 60 and 70 mg/dL was 68% and 87%, respectively, and at 180 and 240 mg/dL were 94% and 92%, respectively.

#### Sensor longevity

**FIG. 1.**
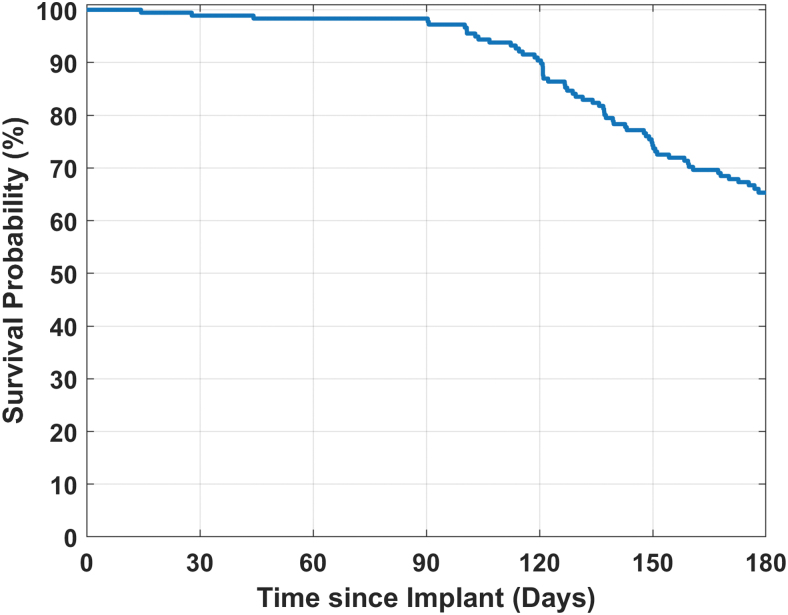
The Kaplan–Meier survival analysis for the primary sensor.

As illustrated in Figure 1, the Kaplan–Meier survival analysis for the primary sensors, the estimated sensor survival probability was 98% on day 90, 90% on day 120, 74% on day 150, and 65% on day 180.

#### Calibration stability

The analysis of calibration duration showed that a calibration duration between 22 and 24 h had a 15%/15% agreement of 83.2% and a 20%/20% agreement of 92.4%. With a duration of 24–26 h, the 15%/15% agreement was 82.3% and 20%/20% was 91.4%. Calibration stability assessment of the primary sensor showed that the 15%/15% metric ranged from 87.5% in the first 2 h after calibration to 82.3% at 26 h after calibration.

The system prompted a median of one calibration per day 56% of the time after day 21.

#### HbA1c

The HbA1c was 7.6% at baseline, 7.2% at 90 days, and 7.3% at day 180.

#### Precision analysis with the primary and the secondary sensors with identical chemistry

Matched pairs were generated during in-clinic sessions, which demonstrated a correlation between the two sensors with the mean Glucose_Sensor1_−Glucose_Sensor2_ difference (±standard deviation) at 0.1 mg/dL (±22.3 mg/dL). In addition, imprecision was measured by PARD and PCV. These data yielded a PARD of 10.1% and PCV of 7.1%, demonstrating high precision.

### The SBA sensor results

#### Subjects

A subgroup of 43 consecutively enrolled subjects in this study received the SBA sensor as their secondary sensor. The SBA subgroup had a mean age of 48.5 years, a mean body mass index of 32 kg/m^2^, and other demographics consistent with the entire cohort.

#### Accuracy

The overall accuracy of the 43 SBA sensors with 12,034 matched glucose pairs is given in Table 4. The overall MARD was 8.5% for the SBA sensor through 180 days, while the 15%/15% analysis was 87.3% and the 20%/20% analysis was 93.9%.

**Table 4.** Continuous Glucose Monitoring System Agreement to Reference Within Yellow Springs Instrument Glucose Ranges Through 180 Days: Sacrificial Boronic Acid Sensor

YSI glucose range (mg/dL)	No. of paired CGM and YSI reference points	Mean percent 15%/15% of reference	Mean percent 20%/20% of reference	MARD*^a^* (%)	Median absolute relative difference (%)
**Overall**	**12,034**	**87.3**	**93.9**	**8.5**	**6.4**
40–60	592	92.6	96.5	7.5	6.0
61–80	1221	89.4	96.8	7.7	6.0
81–180	5067	84.6	92.0	8.6	6.7
181–300	3300	87.5	94.2	7.4	5.5
301–350	1457	90.6	95.9	6.9	5.3
351–400	372	94.1	97.0	6.4	4.7

^a^ MAD (mg/dL) was calculated for glucose values ≤80 mg/dL.

Table 5 shows data for the SBA sensors across the sensor lifetime from day 1 to 180. The lowest accuracy performance was on day 1.

**Table 5.** Continuous Glucose Monitoring System Accuracy by Visit: Sacrificial Boronic Acid Sensor

Day no.	No. of paired CGM-YSI	Mean percent 15%/15% of reference	Mean percent 20%/20% of reference	MARD*^a^* (%)	Median absolute relative difference (%)
Day 1	1203	78.6	87.4	11.2	9.3
Day 7	792	81.9	88.0	10.0	5.8
Day 14	404	87.4	95.0	7.4	4.9
Day 22	1436	88.9	95.7	8.4	6.5
Day 30	1523	85.8	93.4	8.2	5.9
Day 60	1365	87.9	94.2	8.6	6.7
Day 90	1418	93.1	97.1	7.0	5.7
Day 120	1195	89.2	96.1	8.4	6.5
Day 150	1285	84.0	91.9	8.8	6.5
Day 180	1413	93.1	98.0	7.4	6.3

^a^ MAD (mg/dL) was calculated for glucose values ≤80 mg/dL.

#### Alert rates

Detection rates confirming a hypoglycemic or hyperglycemic event showed the confirmed event detection rate at the alert setting of 60 and 70 mg/dL was 90% and 94%, respectively, and at 180 and 240 mg/dL was 99% and 98%, respectively. The true alert rate at 60 and 70 mg/dL was 73% and 84%, respectively, and at 180 and 240 mg/dL was 93% and 91%, respectively.

#### Sensor longevity

**FIG. 2.**
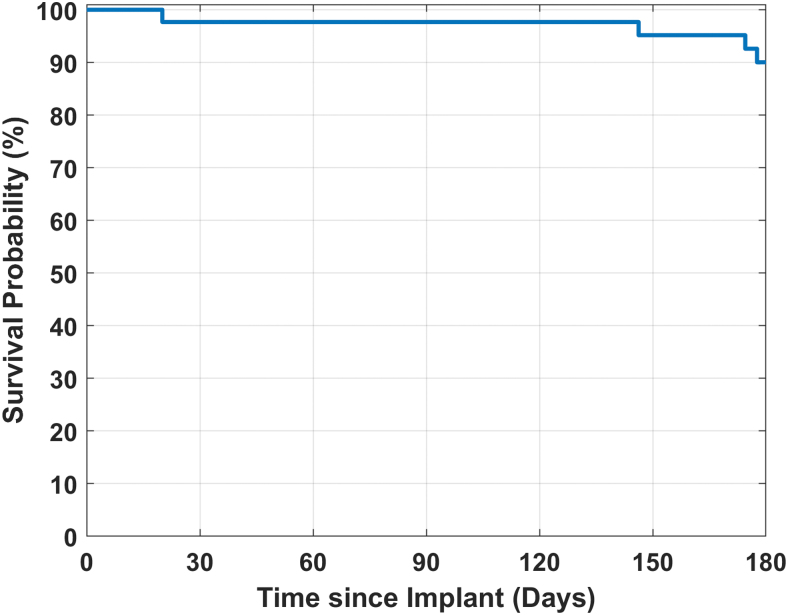
The Kaplan–Meier survival analysis for the SBA sensor. SBA, sacrificial boronic acid.

Figure 2 shows the Kaplan–Meier survival analysis for the 43 SBA sensors. The sensor survival was 96% on day 30, 60, 90, and 120; 94% on day 150; and 90% on day 180.

#### Calibration stability

The analysis of calibration duration showed that a calibration duration between 22 and 24 h had a 15%/15% agreement of 89.7% and 20%/20% agreement of 96.2%. With a duration of 24–26 h, the 15%/15% agreement was 93.5% and 20%/20% agreement was of 98.2%. Calibration stability assessment of the SBA sensor showed that the 15%/15% metric ranged from 88.8% in the first 2 h after calibration to 93.5% at 26 h after calibration.

The SBA sensor system prompted a median of one calibration per day 62% of the time after day 21.

### Safety

There were no SAEs related to the device or insertion/removal procedures. There were no unanticipated AEs and no unanticipated adverse device effects. The majority of device- or insertion/removal procedure-related AEs was mild in severity and resolved shortly after sensor insertion or removal.

In the primary sensor arm, 59 AEs in 37 subjects (20.4%, Supplementary Table S1) were adjudicated to be device or insertion/removal procedure related or possibly related through day 180 and postsensor removal. The most reported related AEs were dermatological in nature (such as skin irritation to the adhesive patch, skin atrophy, hypopigmentation, and infection), affecting 11.6% of the subjects. The next most reported related AEs were hematologic in nature (such as bruising and bleeding) affecting 7.7% of the subjects. Finally, 3.9% of subjects experienced related AEs categorized as neurological in nature (such as pain). All primary sensors were removed on first attempt in all 181 subjects.

In the subjects with the SBA sensor, 17 AEs in 10 subjects (23.3%) were adjudicated to be device or insertion/removal procedure related or possibly related through day 180 and postsensor removal. The AEs reported with the SBA sensor were similar in nature to the AEs associated with the primary sensor. Not all SBA sensors were removed at the end of the 180-day period, since 30 of the 43 subjects consented to participate in a 365-day feasibility extension phase of the protocol. All 13 SBA sensors were successfully removed at the first attempt after the day 180 visit.

In the study overall, there were 279 sensor insertions (85 single sensors +96 dual sensors +2 replacements), resulting in 558 insertion/removal procedures. There were two infections (one associated with an SBA sensor) observed at insertion or removal, resulting in an incision infection rate in 1.1% of subjects or in 0.36% of the total insertion and removal procedures performed.

## Discussion

The primary objectives of the PROMISE study were to evaluate the accuracy and safety of the next-generation Eversense CGM system over a 180-day period across the glucose ranges of 40–400 mg/dL. The effectiveness measurements for the primary sensors in all subjects, based on 49,613 matched pairs, showed that 85.6% of CGM readings were within 15%/15% of the YSI values and 92.9% of the CGM readings were within 20%/20% of the YSI values across all glucose ranges, including hypoglycemia and hyperglycemia. The MARD observed over the 180-day period was 9.1%, with a mean absolute difference (MAD) in the hypoglycemic range of 9.4% for glucose levels 40–60 mg/dL and 8.8% for values 61–80 mg/dL.

The detection of hypoglycemia (at 70 mg/dL) and hyperglycemia (at 180 mg/dL) with confirmed events was in the 93%–99% range and the precision measure (PARD) for the primary sensor and its identical match were 10.1%. These results are similar to or better than accuracy measurements of other commercially available CGM systems^14–16^ and for a much longer duration than any of these other devices. The subset of SBA sensors had 87.3% of CGM readings within 15%/15% of the YSI values and 93.9% of CGM readings were within 20%/20%. The MARD was 8.5% over the glucose range of 40–400 mg/dL during the entire 180 days and the MAD was below 8% in the hypoglycemic range. These results for the SBA sensors, although similar to the results from the primary sensors, were improved.

To enable a long-term implanted fluorescent sensor to survive 180 days or longer with appropriate accuracy, it is critical to decrease the impact of oxidation on the sensing surface.^17,18^ During the development of the Eversense CGM system, in-vivo clinical testing showed that the boronate recognition element had been oxidized as a result of ROS, particularly hydrogen peroxide.^18^ This natural inflammatory response to the sensor caused de-boronation of the indicator molecule and impacted glucose binding. To counteract that effect, nanoparticle metallic platinum was placed onto the porous surface of the hydrogel. In the original sensors, this had a protective effect on the sensor signal, extending its useful lifetime up to 3 months. However, ongoing research showed that modification of the sensor hydrogel indicator surface by adding VPBA could be of further benefit to reduce sensor oxidation by ROS, as was done in the SBA sensors. The 43 SBA sensors had a mean 90% survival and a MARD of 8.5%. Therefore, the addition of SBA to the sensor chemistry increased sensor longevity with improved sensor accuracy and without compromising safety.

While factory calibration might be possible for transcutaneous sensors lasting up to 2 weeks, a long-term implantable sensor lasting up to 180 days, essentially 12 times longer, requires calibration. The new calibration algorithm employed in the PROMISE study, which determines the change in sensitivity from indicator oxidation, showed that calibration frequency could be reduced to one time per day after day 21 to 54% of the time, enabling the next-generation Eversense CGM system to reduce the burden of implantable CGM usage. With the SBA sensors, there was an increase in the percentage of time with one calibration to almost two-thirds of the time.

There were no unanticipated AE and no device- or insertion/removal procedure-related SAEs in the study overall. As a result, the PROMISE study provided further evidence of the safety of the Eversense 180 CGM System, which has been demonstrated in other clinical studies^7–9^ and real-world evaluation.^10,19,20^ Many of the AEs associated with the Eversense 180 CGM system, including with SBA sensors, are common to all CGM systems. However, due to the unique requirement for a minor office-based procedure to insert and remove the sensor, the insertion/removal procedure-related AEs in the PROMISE study, including with the SBA sensor, were minor and similar to that observed in the 90-day US pivotal studies^8,9^ and in studies evaluating the present EU Eversense system.^19,21^

The data from the PROMISE study support the next-generation Eversense CGM system, with reduced calibration enabling a single calibration point to be entered on about half of the days of system wear. The next-generation Eversense CGM, which is currently under consideration by the Food and Drug Administration, with its many unique features, including its implantation by a health care provider, its long duration, its removable transmitter that has vibratory alerts, and being held in place with a mild silicone-based adhesive, may be an option in the future pending regulatory approval for patients anticipated to benefit from real-time CGM.

## Conclusion

The PROMISE study, evaluating the next-generation long-term implantable Eversense CGM system, showed that both the primary and SBA sensors were safe and accurate lasting up to 180 days. The next-generation CGM system has an improved calibration algorithm allowing for a single calibration point per day on about half of the days of system wear.

## Supplementary Material

Supplemental data
