# Supplementary material for: Evaluation of Accuracy and Safety of the Next-Generation Up to 180-Day Long-Term Implantable Eversense Continuous Glucose Monitoring System: The PROMISE Study
Source: Diabetes Technol Ther. 2022 Jan 31;24(2):84–92. doi: 10.1089/dia.2021.0182 (PMC8817689; doi:10.1089/dia.2021.0182)
Supplement: Supplemental data [file Supp_TableS1.docx]

**Supplemental Table 1. Device or Insertion/Removal-Related Adverse Events: All Participants**

| **Event Physiologic System** | **Number of Events** | **Number of Subjects (% of Subjects)** |
| --- | --- | --- |
|  | 59 | 37 (20.4) |
| Dermatological  Skin irritation, adhesive patch location (including erythema, pruritus, rash, contact dermatitis)  Skin atrophy  Hypopigmentation  Infection (insertion/removal site)  Infection (under adhesive patch)  Contact dermatitis due to drape adhesive  Erythema, insertion site  Seroma, removal site | 27  13  4  4  2  1  1  1  1 | 21 (11.6)  9 (5.0)  4 (2.2)  3 (1.7)  2 (1.1)  1 (0.6)  1 (0.6)  1 (0.6)  1 (0.6) |
| Hematologic Immunologic  Bruise (1 w/ serosanguinous drainage)  Bleeding | 22  19  3 | 14 (7.7)  11 (6.1)  3 (1.7) |
| Neurological  Pain  Arm Numbness  Tremor | 9  7  1  1 | 7 (3.9)  6 (3.3)  1 (0.6)  1 (0.6) |
| Other  Steristrips did not hold | 1  1 | 1 (0.6)  1 (0.6) |
